# Two Independently Comparative Transcriptome Analyses of Hemocytes Provide New Insights into Understanding the Disease-Resistant Characteristics of Shrimp against *Vibrio* Infection

**DOI:** 10.3390/biology12070977

**Published:** 2023-07-10

**Authors:** Shihao Li, Keke Zhang, Wenran Du, Fuhua Li

**Affiliations:** 1 CAS and Shandong Province Key Laboratory of Experimental Marine Biology, Institute of Oceanology, Chinese Academy of Sciences, Qingdao 266071, Chinafhli@qdio.ac.cn (F.L.); 2 Center for Ocean Mega-Science, Chinese Academy of Sciences, Qingdao 266071, China; 3 University of Chinese Academy of Sciences, Beijing 100049, China; 4 School of Marine Science and Engineering, Qingdao Agricultural University, Qingdao 266109, China; 5 The Innovation of Seed Design, Chinese Academy of Sciences, Wuhan 430072, China

**Keywords:** penaeid shrimp, hemocyte, disease-resistant, *Vibrio parahaemolyticus*

## Abstract

**Simple Summary:**

Identification of disease-resistant genes and their application for disease-resistance breeding is an important way to solve disease problems in shrimp aquaculture. Although some transcriptomics studies have been performed to identify genes related to pathogen infection and host defense, there is still a lack of deep understanding of the host-resistant characteristics against *V. parahaemolyticus* infection. The present study established a method to obtain hemocytes from shrimp with clear resistant abilities against *V. parahaemolyticus* infection. We also proposed a strategy to identify key biological processes and genes related to disease resistance based on the analysis of the present transcriptome data.

**Abstract:**

*Vibrio parahaemolyticus* carrying plasmid encoding toxins PirA and PirB is one of the causative agents leading to the severe disease of AHPND in shrimp aquaculture. However, there is a lack of deep understanding of the host-resistant characteristics against *V. parahaemolyticus* infection. Here, we established a method to obtain hemocytes from shrimp with different *V. parahaemolyticus*-resistant abilities and performed comparative transcriptome analysis on the expression profiles at the background level of hemocytes from shrimp in two independent populations. Principal component analysis and sample clustering results showed that samples from the same population had a closer relationship than that from shrimp with similar disease-resistant abilities. DEGs analysis revealed that the number of DEGs between two populations was much more than that between *V. parahaemolyticus*-resistant and susceptible shrimp. A total of 31 DEGs and 5 DEGs were identified from the comparison between *V. parahaemolyticus*-resistant and susceptible shrimp from populations 1 and 2, respectively. DEGs from population 1 were mainly cytoskeleton-related genes, metabolic related genes, and immune related genes. Although there was no DEGs overlap between two comparisons, DEGs from population 2 also included genes related to cytoskeleton and metabolism. The data suggest that these biological processes play important roles in disease resistance, and they could be focused by comprehensive analysis of multiple omics data. A new strategy for screening key biological processes and genes related to disease resistance was proposed based on the present study.

## 1. Introduction

Shrimp is one of the most important aquatic animals in aquaculture. However, frequent diseases caused by bacteria, viruses, and parasites always lead to severe economic losses, which greatly hinder the healthy development of shrimp aquaculture. AHPND, previously called early mortality syndrome, is one of the most serious diseases caused by bacteria [1]. The agents causing AHPND are *Vibrio* species that contain a plasmid encoding the lethal toxins PirA and PirB [2]. *Vp*_AHPND_, *Vibrio parahaemolyticus* carrying the plasmid, is one of the causative agents leading to AHPND in shrimp aquaculture [3]. 

Although the hepatopancreas is the main target tissue of the bacteria, *V. parahaemolyticus* tends to cause systemic responses in the host. In the stomach of *Penaeus monodon*, many immunity-related genes including antimicrobial peptides, prophenoloxidase system, proteinase/proteinase inhibitors, and signal transduction pathways were responsive to *V. parahaemolyticus* infection [4]. In the hemocytes of *L. vannamei*, genes related to carbohydrate metabolism, lipid metabolism, amino acid metabolism, and cell growth and anti-apoptosis were significantly changed after *V. parahaemolyticus* infection [5]. Another study targeting the hemocytes of *L. vannamei* identified many immunity-related genes such as crustin, anti-lipopolysaccharide factor, serpin 3, and C-type lectin responsive to *V. parahaemolyticus* infection [6]. In the hepatopancreas of *L. vannamei*, genes related to programmed cell death, carbohydrate metabolism, and biological adhesion were up-regulated after *V. parahaemolyticus* infection [7]. Another study revealed that the hepatopancreas of *L. vannamei* responded to *V. parahaemolyticus* infection by activating glucose metabolism, energy metabolism, amino acid metabolism, nucleic acid synthesis, as well as HIF-1 signaling pathway, PI3K/Akt signaling pathway, and NF-KappaB signaling pathway [8]. These data provide useful information for our understanding of the overall responses of the host against *V. parahaemolyticus* infection.

Due to the different genetic backgrounds, shrimp exhibit distinct disease-resistant abilities to *V. parahaemolyticus*. The variation of disease-resistant ability could be reflected by differential expression of genes at the background level. In *P. vannamei*, several genes including chymotrypsin A, serine protease, crustin-P, and prophenol oxidase activation system 2 showed differential expression levels between *Vp*_AHPND_-tolerant and susceptible populations [9]. A transcriptomic comparison between three *Vp*_AHPND_-resistant and three susceptible families identified 489 DEGs in *L. vannamei,* and 19 tested genes also showed different expression profiles in their offsprings [10]. Although few common genes were reported in different studies due to the distinct genetic backgrounds, these identified genes could be used as potential biomarkers for understanding the *Vp*_AHPND_ resistance of shrimp. However, there is still a lack of effective strategies to find key genes and regulatory processes that are used for host defense again *Vp*_AHPND_ infection.

As the main immunity-related tissue, hemocytes play important roles in host defense against pathogen infection. However, the identification of genes in hemocytes related to *Vp*_AHPND_ resistance has not been reported. In the present study, the comparative transcriptome analysis was performed on two independent populations. The transcriptional expression profiles were analyzed at the background level of hemocytes from shrimp with different resistant abilities against *Vp*_AHPND_ infection in each population. Some genes related to *Vp*_AHPND_ resistance were identified in shrimp hemocytes while no common DEG existed between two comparisons. A strategy was proposed based on the present study for further identification of key genes and regulatory processes related to shrimp disease resistance.

## 2. Materials and Methods

### 2.1. Experimental Animals

Two batches of *Litopenaeus vannamei* purchased from Rizhao breeding farm in Shandong were used for two independent experiments. Before experiments, the shrimp were cultured in aerated seawater laboratory aquaculture tank at 26 °C for seven days. The shrimp weights were 10 ± 0.5 g in the first batch (population 1) and 19.5 ± 1 g in the second batch (population 2). The hepatopancreas from three individuals was dissected, mixed, and ground. A total of 0.18 g tissue sample was homogenized in 1 mL PBS and then spread on the TCBS solid medium plate. The plate was put in the 30 °C incubator overnight to confirm whether the shrimp were free of *Vibrio* pathogens. Three biological replicates were performed for each population.

### 2.2. VIE Fluorescent Labeling and Hemolymph Collection

The VIE distinguished with eight different colors (red, pink, orange, brown, white, purple, green, yellow) was injected into each shrimp in the third segment with different color fluorescence. A total of 80 and 70 individuals were used in two experiments, respectively. Then, the shrimp were cultured in aerated seawater at 25 ± 1 °C for seven days and fed with artificial food twice a day. The seawater was changed every day. After one week, 200 μL hemolymph was exsanguinated from each shrimp with a sterile syringe containing an equal volume of ice-cold anticoagulant (27 mM trisodium citrate, 336 mM sodium chloride, 115 mM glucose, 9 mM Na_2_EDTA·2H_2_O, pH 7.4). The hemocytes were separated by centrifugation at 1000 *g* for 10 min and stored in −80 °C. Then, all the shrimp were returned to the laboratory tanks.

### 2.3. V. parahaemolyticus Immersion and Hepatopancreas Collection

One week after exsanguination, the shrimp were infected with *V. parahaemolyticus* AG01, a strain isolated in our lab [11]. During *V. parahaemolyticus* infection, shrimp were cultured in the same condition while the seawater was not changed for two days. The bacteria strain was cultured in tryptic soy broth with 2% sodium chloride liquid media. The cultured bacteria were further checked by positive PCR amplification of *PirA^vp^* and *PirB^vp^* genes. Bacterial titer was counted with a hemocytometer under a light microscope. The soak infection doses in two batches of shrimp were set at the lethal concentration 50, 2.5 × 10^6^ CFU/mL and 5 × 10^6^ CFU/mL, respectively. The mortality was continuously recorded for two days. The hepatopancreas of moribund shrimp and survival shrimp after 48 h post infection was collected and stored at −80 °C. In addition, the hepatopancreas of moribund shrimp was also collected for pathogen detection. The tissue was treated as described in Section 2.1, and the *Vibrio* strain grown on the TCBS plate was collected as PCR template for 16S rDNA amplification and Sanger sequencing. The *Vibrio* strain was confirmed as *V. parahaemolyticus* before further analysis.

### 2.4. DNA Extraction and Bacteria Load Detection

The DNA from the hepatopancreas was extracted using TIANGEN Plant Genomic DNA Extraction Kit according to the manufacturer’s instruction. The concentration and purity of DNA were determined using NanoDrop 2000 (Thermo Fisher Scientific, Waltham, MA, USA) by coagulation and 1% gel electrophoresis. PirA copy number was estimated by Ependorf MasterCycler EP Realplex (Ependorff, Germany) using TaqMan Assay. A forward primer PirA-F and reverse primer PirA-R were used to amplify a 284 bp fragment of PirA gene, with a TaqMan hydrolysis probe 5′-FAM-CCGCCAGCCATAAATGGCGCACC-BHQ1-3′). The amplification program consisted of one cycle of contamination digestion at 37 °C for 2 min, one cycle of pre-denaturation at 95 °C for 5 min, and 45 cycles of denaturation at 95 °C for 10 s and extension at 60 °C for 30 s. A standard curve was obtained using serial dilutions of plasmid PirA (full-length ORF of PirA gene of *V. parahaemolyticus* was cloned into pUC57 vector), which was used to quantify the *V. parahaemolyticus* copy number. Each assay was carried out in four parallels. Therefore, all shrimp were divided into resistant group and susceptible group (9 hpi, 12 hpi, and the surviving individual) according to the time of death and *Vibrio* loads.

### 2.5. RNA Extraction and Transcriptome Sequencing

Based on the death and survival data and *Vibrio* load data, all shrimp were divided into *V. parahaemolyticus*-resistant and susceptible individuals. The collected hemocytes of *V. parahaemolyticus*-resistant and susceptible shrimp were mixed, respectively. For population 1, three biological replicates were prepared for resistant and susceptible shrimp. The samples were designated as HcS1-1, HcS1-2, and HcS1-3 for resistant shrimp and designated as HcD1-1, HcD1-2, and HcD1-3 for susceptible shrimp. For population 2, four replicates were prepared for resistant and susceptible shrimp. The samples were designated as HcS2-1, HcS2-2, HcS2-3, and HcS2-4 for resistant shrimp and designated as HcD2-1, HcD2-2, HcD2-3, and HcD2-4 for susceptible shrimp. The total RNA of hemocytes was extracted by RNAiso Plus (TaKaRa, Kyoto, Japan) according to the manufacturers’ protocols. The total RNA was qualified by 1% agarose gel electrophoresis and quantified by NanoDrop 2000 (Thermo Fisher Scientific, Waltham, MA, USA). About 1 μg total RNA from each sample was pre-treated with gDNA Eraser to remove genomic DNA according to the manufactures’ instruction (TaKaRa, Kyoto, Japan). Fragmentation buffer was used to break the mRNA into short fragments. The first cDNA strand was synthesized using random hexamers, followed by the addition of buffer, dNTPs, RNase H, and DNA polymerase I to synthesize the second cDNA strand. Poly(A) was added to connect to the sequencing adaptor. Finally, the Illumina HiSeq2500 platform was used to sequence the library at Guangzhou Gene Denovo Biotech Co., Ltd. (Guangzhou, China).

### 2.6. Clean Data Mapping and Annotations

In order to ensure the data quality, the clean reads obtained from the preliminary filtering were subjected to more stringent filtering. High-quality clean reads were obtained by eliminating all the reads containing the A base and more than 10% of the reads containing N. The clean reads were mapped to the reference genome of *L. vannamei* by HISAT2.2.4 [12]. The mapped reads were assembled using StringTie v 1.3.1 [13] in a reference-based approach. The reconstructed transcripts were aligned to the reference genome. Annotation was carried out by blast against NR, KEGG, COG, and Swiss-Prot databases.

### 2.7. Differential Expression and Enrichment Analysis

The DEGs in hemocytes between *V. parahaemolyticus*-resistant and susceptible shrimp were identified by calculating the FPKM value. DEGs were analyzed using the edgeR package (http://www.rproject.org/, 10 January 2023), with the parameter of FDR < 0.05 and the absolute fold change ≥ 2. The correlation analysis between samples was carried out among samples to ensure the reliability of experimental data. 

The GO function and KEGG pathway enrichment analyses of DEGs were performed using the online OmicShare tools (http://www.omicshare.com/tools, 12 January 2023). The Q value < 0.05 was considered statistically significant.

### 2.8. Quantitative Real-Time PCR

The validation was conducted by qRT-PCR using the RNA prepared for RNA-seq. The first strand of cDNA was reverse transcribed from 1 μg RNA with PrimeScript™ RT Reagent Kit with gDNA Eraser (TaKaRa, Kyoto, Japan). The products were diluted by 20-fold with nuclease-free water. Primers for qRT-PCR (Table S1) were designed for six DEGs from population 1 and all five DEGs from population 2 using Primer Premier 5.0 software. Additionally, 18s rRNA was used as an internal control to standardize the expression levels. The qRT-PCR was performed using THUNDERBIRD^®^ SYBR^®^ qPCR Mix on the Eppendorf Mastercycler ep realplex (Eppendorf, Germany) in a total volume of 10 μL, containing 5 μL qPCR mix, 1 μL diluted cDNA, 0.3 μL each of forward and reverse primers, and 3.4 μL nuclease-free water. The amplification programs were set as follows: 95 °C for 2 min; 40 cycles of 95 °C for 15 s, annealing temperature for 15 s, and 72 °C for 30 s; and followed by a melting curve. Three technical replicates were set for each sample. The expression analysis was calculated by 2^∆∆ct^ and expression profiles of DEGs were shown with log2 fold change values.

## 3. Results

### 3.1. The Loads of V. parahaemolyticus in Hepatopancreas of Infected Shrimp

**Figure 1:**
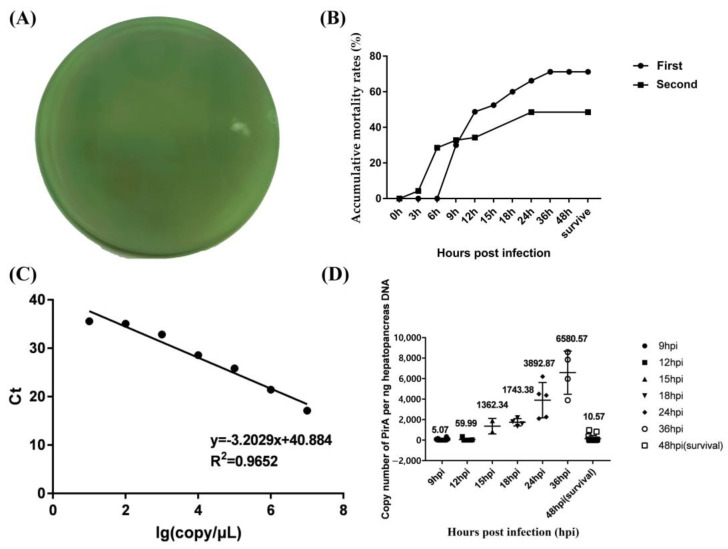
The load of *V. parahaemolyticus* in hepatopancreas of infected shrimp from two populations. (**A**) shows the detection of *Vibrio* species in hepatopancreas of shrimp used in experiments. (**B**) shows the accumulative mortality rates of shrimp in two experiments. (**C**) shows the standard curve equation of PirA^Vp^. (**D**) shows the copy number of *V. parahaemolyticus* per ng hepatopancreas DNA at different hours post infection (hpi). Survival shrimp after 48 hpi were shown by 48 hpi (survival).

Detection of *Vibrio* pathogens showed that there was no strain on the TCBS plate (Figure 1A), suggesting that the shrimp used in experiments were free of the specific pathogens. The accumulative mortality rates for two populations were 71.25% and 48.57%, respectively (Figure 1B). The standard curve equation of PirA^Vp^ was established as y = −3.2029x + 40.884, where x represented the log value of *Vibrio* copy number per μL standard plasmid PirA, and y represented the Ct value. The correlation coefficient was 0.9652, which showed a good linear relationship between the concentration of standard substance and the Ct value (Figure 1C).

As shown in Figure 1D, the loads of *V. parahaemolyticus* in hepatopancreas of shrimp died at 9 hpi and 12 hpi were very low, which were 5.07 and 59.99 copies per ng hepatopancreas DNA. The loads of *V. parahaemolyticus* were high in hepatopancreas of shrimp died at 15 hpi, 18 hpi, 24 hpi, and 36 hpi, all of which were more than 1000 copies per ng of hepatopancreas DNA. The load of *V. parahaemolyticus* in hepatopancreas of survival shrimp (still alive at 48 hpi) was 10.57 copies per ng of hepatopancreas DNA. According to the death time and the load of *V. parahaemolyticus* in hepatopancreas, shrimp that died at 9 hpi and 12 hpi were deemed as susceptible individuals, and those with survival after 48 hpi were deemed as resistant individuals. Hemocytes from *V. parahaemolyticus*-resistant and susceptible shrimp in two populations were used for independent comparative transcriptome analysis.

### 3.2. The Correlation of the Transcriptome Data from All Samples

**Figure 2:**
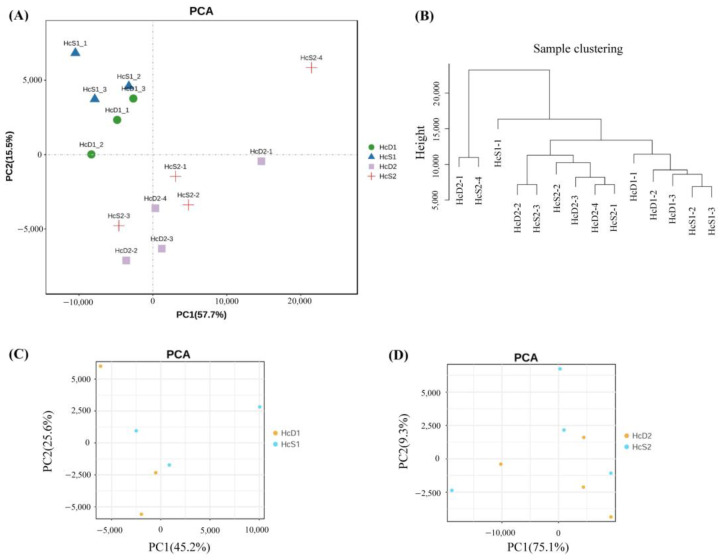
Sample relationship analyzed by PCA and sample clustering analysis. (**A**,**B**) shows the results of PCA and clustering analysis for all samples from two populations. (**C**,**D**) shows the results of PCA for samples from population 1 and 2, respectively.

The relationship of all samples from two populations were analyzed by PCA and sample clustering methods. The results showed that samples from the same population had a close relationship (Figure 2A,B). However, the difference between *V. parahaemolyticus*-resistant and susceptible shrimp was small in each population (Figure 2C,D). The results suggest that shrimp from the same population, even with different *V. parahaemolyticus*-resistant abilities, have more similar transcriptome profiles than those from different populations.

### 3.3. DEGs between Two Populations and between V. parahaemolyticus Resistant and Susceptible Shrimp

**Figure 3:**
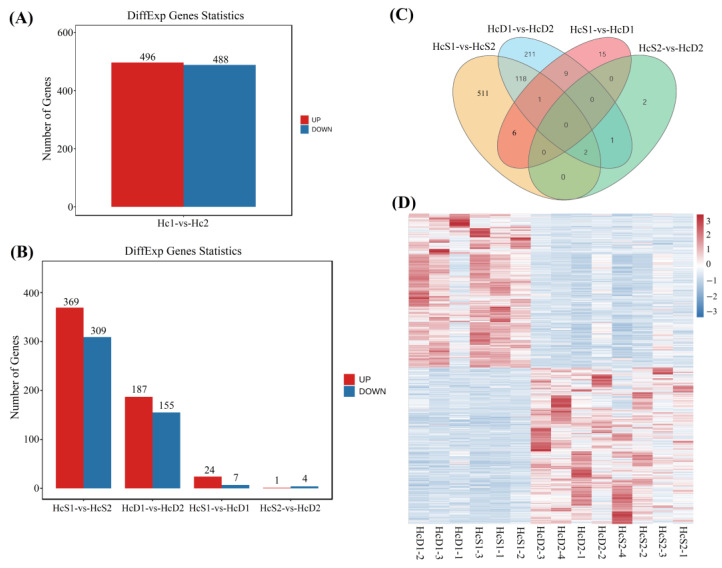
Identification of DEGs between different comparisons. (**A**) shows DEGs between population 1 and 2. (**B**) shows DEGs from comparisons HcS1-vs-HcS2, HcD1-vs-HcD2, HcS1-vs-HcD1, and HcS2-vs-HcD2. (**C**) shows the result of Venn analysis of DEGs from different comparisons. (**D**) shows the heatmap of DEGs between two populations.

Corresponding to the sample relationship, much more DEGs were identified between two populations than those between *V. parahaemolyticus*-resistant and susceptible shrimp in each population. Between two populations, there were a total of 984 DEGs, including 496 up-regulated DEGs and 488 down-regulated DEGs in the hemocytes from population 2 (Figure 3A). There were 678 DEGs between the *V. parahaemolyticus*-resistant shrimp from population 1 and 2 (Figure 3B, HcS1-vs-HcS2). There were 342 DEGs between the *V. parahaemolyticus*-susceptible shrimp from population 1 and 2 (Figure 3B, HcD1-vs-HcD2). 

Very few DEGs were identified between *V. parahaemolyticus*-resistant and susceptible shrimp. There were 31 DEGs and 5 DEGs between *V. parahaemolyticus*-resistant and susceptible shrimp in population 1 and population 2, respectively (Figure 3B, HcS1-vs-HcD1, HcS2-vs-HcD2; Tables S2 and S3). 

A total of 121 DEGs were overlapping between HcS1-vs-HcS2 and HcD1-vs-HcD2 (Figure 3C). The expression trends of these DEGs showed obvious differences between two populations (Figure 3D). However, there were no overlapping DEGs between HcS1-vs-HcD1 and HcS2-vs-HcD2 (Figure 3C), suggesting that distinct genes contribute to the disease-resistant ability of shrimp against *V. parahaemolyticus* infection in two populations.

### 3.4. Enriched GO Items and KEGG Pathways between V. parahaemolyticus-Resistant and Susceptible Shrimp

**Figure 4:**
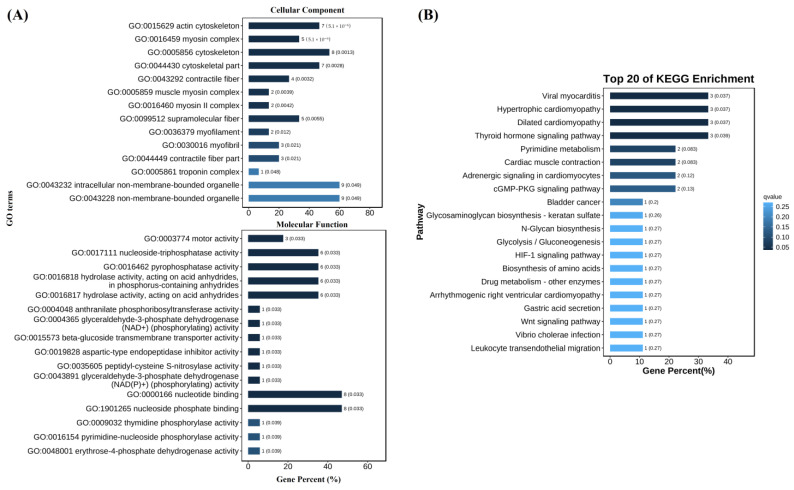
GO (**A**) and KEGG (**B**) enrichment of DEGs between *V. parahaemolyticus*-resistant and susceptible shrimp in population 1.

GO and KEGG enrichment results showed that some DEGs in population 1 were enriched in GO items and KEGG pathways. The enrichment items in cell composition were mainly “actin cytoskeleton”, “myosin complex”, “cytoskeleton”, etc. The enriched items in molecular function were “motor activity”, “nucleoside-triphosphatase activity”, “pyrophosphatase activity”, etc. (Figure 4A). The enriched KEGG pathways included “viral myocarditis”, “hypertrophic cardiomyopathy”, “dilated cardiomyopathy” and “thyroid hormone signaling pathway” (Figure 4B). There were no GO item and KEGG pathway enriched for DEGs in population 2 because only five DEGs were identified.

### 3.5. Detailed Analysis of DEGs between V. parahaemolyticus-Resistant and Susceptible Shrimp

Among the 31 DEGs in population 1, 19 had functional annotations (Table 1). Most of these DEGs exhibited higher expression levels in *V. parahaemolyticus*-susceptible shrimp. DEGs encoded cytoskeleton-related proteins, including troponin, myosin, septin-4, and actin, as well as immune and signal transduction-related genes, such as immunity-associated nucleotide-binding protein 13-like, E3 ubiquitin-protein ligase TRIM32, and MAM and LDL-receptor class A domain-containing protein 2-like, which had higher expression levels in *V. parahaemolyticus* susceptible shrimp. Sugar metabolism-related genes including glyceraldehyde-3-phosphate-dehydrogenase and alpha-(1,6)-fucosyltransferase-like were also highly expressed in *V. parahaemolyticus*-susceptible shrimp.

In population 2, one cytoskeleton-related genes-encoding tubulin α-3 was also highly expressed in *V. parahaemolyticus*-susceptible shrimp. The other four DEGs, including dihydropyrimidinase-like isoform X3, prohibitin, iroquois-class homeodomain protein IRX-2-like, and diacylglycerol kinase 1, were all highly expressed in *V. parahaemolyticus*-resistant shrimp (Table 2).

**Figure 5:**
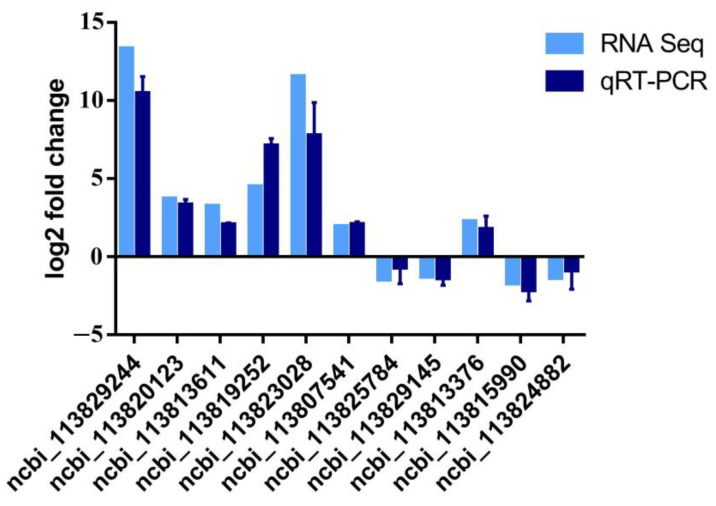
Validation of expression profiles in transcriptome data by qRT-PCR for 11 DEGs from two populations. DEGs are shown with the NCBI accession numbers.

In order to validate the expression of DEGs in the transcriptome, we compared the expression trends from transcriptome data and qRT-PCR data for six DEGs from population 1 and five DEGs from population 2. The results showed that all genes had similar expression changes between transcriptome data and qRT-PCR data (Figure 5), suggesting that the differential expression analysis in transcriptome data was credible.

## 4. Discussion

In order to screen genes in shrimp hemocytes related to *V. parahaemolyticus* resistance, the present study established a method to collect hemocytes from shrimp with different resistant abilities against *V. parahaemolyticus* infection. The results showed that shrimp more susceptible to *V. parahaemolyticus* infection died early with low pathogen load. When shrimp exhibited higher resistant ability against *V. parahaemolyticus* infection, they could suffer from higher pathogen load and stayed alive for a longer time. Shrimp with the highest resistant ability against *V. parahaemolyticus* infection could clear the pathogen in hepatopancreas after a longer time (48 h) post infection. Shrimp families with distinct resistances against specific pathogens were usually constructed, while individuals in the same family always exhibited different disease-resistant abilities. The method used in the present study could collect hemocytes from shrimp before pathogen infection with clear pathogen resistance information. 

Free of *Vibrio* species ensured that the transcriptome profiles of shrimp hemocytes could represent the background expression levels of genes that were not influenced by specific pathogens. Immune challenge test showed that two populations had distinct resistant abilities against *V. parahaemolyticus* infection. This might be attributed to the different genetic backgrounds and animal sizes of the two populations. Nevertheless, different individuals in each population exhibited various disease-resistant abilities. Therefore, two independent transcriptome analyses on hemocytes of shrimp with different *V. parahaemolyticus*-resistant abilities were carried out to identify common genes related to the biological trait. However, the identified DEGs in two populations shared no overlap. The result was comparable with some transcriptome studies on shrimp hepatopancreas, which also identified rare common genes related to *V. parahaemolyticus* resistance [9,10]. It might be caused by the different genetic backgrounds of shrimp. In other words, different populations might employ distinct genes to resist *V. parahaemolyticus* infection. This brings difficulties for the identification of key genes or regulatory processes related to specific pathogens.

Although there was no overlap between DEGs identified from the two populations, many DEGs were reported relevant to immunity or pathogen infection. The cytoskeleton has four main components including actin, microtubules, intermediate filaments, and septins, which all have important functions in cell-autonomous immunity [14]. An E3 ubiquitin-protein ligase TRIM32 gene was crucial under oxidase stress and during *Vibrio* infection in *L. vannamei* [15]. In intestine, the MAM and LDL-receptor class A domain-containing protein could regulate enterohepatic bile acid signaling, while bile acids are vital factors in mucosal immunity and inflammation [16,17]. Glyceraldehyde-3-phosphate-dehydrogenase is the key enzyme in the glycolytic pathway, which benefits viral replication during WSSV in shrimp [18]. These genes were differentially expressed in hemocytes between *V. parahaemolyticus*-resistant and susceptible shrimp in population 1, indicating that they might contribute to the shrimp resistance against *V. parahaemolyticus* infection.

Even if the DEGs in population 2 were totally different than those in population 1, they were also related to immunity and pathogen infection. In the central nervous system, dihydropyrimidinase-like 3 could regulate the inflammatory response of activated immune cells microglia, which respond to infection and inflammation by producing cytokines and phagocytosing cell debris and pathogens [19]. In *Fenneropenaeus chinensis*, two prohibiting genes played important roles in hemocytes during WSSV infection [20]. Diacylglycerol kinase 1 is responsible for the ATP-dependent phosphorylation of diacylglycerol to phosphatidic acid, both of which are crucial second messengers involved in many immune processes such as regulating Toll-like receptor-induced cytokine production [21]. Tubulin alpha-3 is the major constituent of microtubules, one component of cytoskeleton. These genes might be responsible for variable resistant abilities of shrimp against *V. parahaemolyticus* infection in population 2.

**Figure 6:**
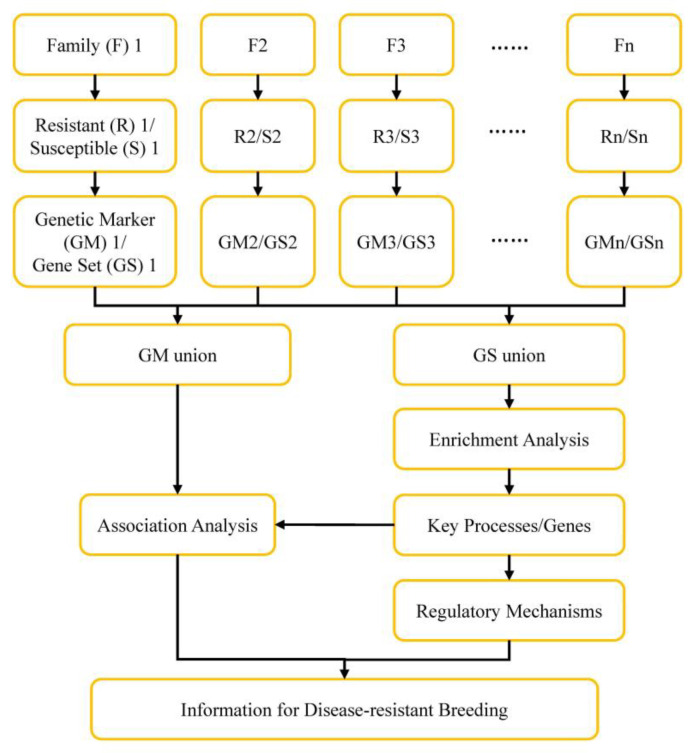
Framework for identification of disease-resistant processes/genes and disease-resistance breeding of shrimp.

Although no common genes related to *V. parahaemolyticus* resistance were identified in hemocytes from two populations, genes in specific GO item-like cytoskeleton could be found in DEGs from both transcriptome data. It means that the key processes or genes related to *V. parahaemolyticus* resistance could be obtained by comprehensive analysis of multiple omics data information. Therefore, a strategy was proposed here to approach this purpose (Figure 6). In this proposal, individuals from the same full sibling family, which have relatively consistent genetic background, will be used to construct *V. parahaemolyticus*-resistant and susceptible hemocytes samples based on the method established in the present study. Differentially expressed genes (Gene Set, GS), as well as genetic makers (GM), will be identified after comparative analysis between *V. parahaemolyticus*-resistant and susceptible samples from each family. The GS and GM information from each family will be pooled respectively to obtain the GS union and GM union. The GS union will be put for GO and KEGG enrichment analyses to identify key processes and genes related to *V. parahaemolyticus* resistance. Further association analysis between these identified key processes and genes and GM union will help to obtain useful genetic markers. The information will provide a basis for understanding regulatory mechanisms of shrimp defense against *V. parahaemolyticus* infection and for disease-resistance breeding.

The proposed strategy was built based on the transcriptome data from two independent shrimp populations. The distinct genetic backgrounds among individuals might limit the identification of DEGs between *V. parahaemolyticus*-resistant and susceptible shrimp. Therefore, we suggested to identify DEGs using shrimp from the same full sibling family in the proposed strategy. A further investigation based on this proposal will be implemented to verify it.

## 5. Conclusions

In the present study, we identified genes related to *V. parahaemolyticus* resistance from hemocytes of two independent shrimp populations. Although no common genes existed in identified DEGs from two populations, there were some DEGs involved in the same biological processes such as cytoskeleton and metabolism. The data suggest that different genes might contribute to the resistant abilities in different shrimp populations, while these genes probably play functions in specific biological processes. Therefore, a strategy was proposed based on the present data and will be verified to identify key processes and genes related to disease resistance.

## Figures and Tables

**Table 1:** DEGs with functional annotations between *V. parahaemolyticus*-resistant and susceptible shrimp in population 1.

Gene ID	HcS1_fpkm	HcD1_fpkm	log2(HcD1/HcS1)	FDR	Description
ncbi_113820920	0.40	0.00	−8.63	0.0376	structural maintenance of chromosomes protein 2-like
ncbi_113815624	0.71	0.08	−3.13	0.0154	vang-like protein 2
MSTRG.1662	5.77	1.38	−2.07	0.0017	APC membrane recruitment protein 1-like
ncbi_113812130	67.07	109.46	0.71	0.0499	4-coumarate—CoA ligase 1 isoform X1
ncbi_113824615	186.25	373.56	1.00	0.0001	spermatogonial stem-cell renewal factor
MSTRG.4583	11.36	42.55	1.91	0.0154	arasin-like protein
ncbi_113807541	51.35	197.85	1.95	0.0397	immune-associated nucleotide-binding protein 13-like
ncbi_113817613	5.39	25.35	2.23	0.0027	E3 ubiquitin-protein ligase TRIM32
ncbi_113824399	82.06	388.92	2.24	0.0023	Septin-4-like protein
ncbi_113813611	0.34	3.21	3.24	0.0373	troponin I
ncbi_113805465	0.66	6.27	3.25	0.0017	myosin light chain 2
ncbi_113822686	0.65	7.58	3.55	0.0001	myosin light chain
ncbi_113820123	5.27	68.89	3.71	0.0000	glyceraldehyde-3-phosphate-dehydrogenase
ncbi_113819252	0.12	2.74	4.50	0.0154	actin 2
ncbi_113816511	0.04	0.95	4.64	0.0035	myosin heavy chain, muscle-like isoform X4
MSTRG.15220	0.18	7.35	5.34	0.0002	Retrovirus-related Pol polyprotein from transposon 297
ncbi_113807016	0.01	0.43	6.44	0.0411	myosin heavy chain, muscle-like isoform X8
ncbi_113823028	0.00	2.97	11.53	0.0000	alpha-(1,6)-fucosyltransferase-like
ncbi_113829244	0.00	10.32	13.33	0.0000	MAM and LDL-receptor class A domain-containing protein 2-like

**Table 2:** DEGs with functional annotations between *V. parahaemolyticus*-resistant and susceptible shrimp in population 2.

Gene ID	HcS2_fpkm	HcD2_fpkm	log2(HcD2/HcS2)	FDR	Description
ncbi_113815990	11.16	3.46	−1.69	0.0184	dihydropyrimidinase-like isoform X3
ncbi_113825784	13.80	5.06	−1.45	0.0473	prohibitin
ncbi_113824882	5.22	2.06	−1.34	0.0184	iroquois-class homeodomain protein IRX-2-like
ncbi_113829145	6.34	2.66	−1.25	0.0402	diacylglycerol kinase 1
ncbi_113813376	1.43	6.81	2.26	0.0142	tubulin alpha-3 chain-like

## Data Availability

The original contributions presented in the study are included in the article/Supplementary Material. The raw data of RNA-Seq have been deposited to NCBI data bank with the accession number PRJNA974583. Further inquiries can be directed to the corresponding authors.

## Supplementary Materials

The following supporting information can be downloaded at: https://www.mdpi.com/article/10.3390/biology12070977/s1, Table S1: Information of primers used in the present study. Table S2: The identified DEGs from population 1. Table S3: The identified DEGs from population 2.

## Abbreviations

The following abbreviations are used in this manuscript:

AHPND	acute hepatopancreatic necrosis disease
DEGs	differentially expressed genes
HIF-1	hypoxia-inducible factor 1
PI3K/Akt	phosphoinositide 3-kinase/protein kinase B
NF-KappaB	nuclear factor kappa-B
VIE	visible implant elastomer
EDTA	ethylene diamine tetraacetic acid
PCR	polymerase chain reaction
cDNA	complementary DNA
NR	non-redundant protein
KEGG	kyoto encyclopedia of genes and genomes
COG	clusters of orthologous genes
FPKM	fragment per kliobase of transcript per million mapped reads
FDR	false discovery rate
GO	gene ontology
PCA	principal component analysis
WSSV	white spot syndrome virus
