# Supplementary material for: Two Independently Comparative Transcriptome Analyses of Hemocytes Provide New Insights into Understanding the Disease-Resistant Characteristics of Shrimp against Vibrio Infection
Source: Biology (Basel). 2023 Jul 10;12(7):977. doi: 10.3390/biology12070977 (PMC10376663; doi:10.3390/biology12070977)
Supplement: Supplementary file 1 [file biology-12-00977-s001.zip › Table S1 Information of primers used in the present study.pdf]

Table S1. Information of primers used in the present study

Gene ID	Gene name	Primer sequence	Product size (bp)	Annealing temperature
/	PirA	F: GGTGACCAGACCTGCTTTGAG<br>R: CCAGCTAGGATAACCGTAACATG	112	60
/	16S rDNA	F: AGAGTTTGATCCTGGCTCAG<br>R: GGTTACCTTGTTACGACTT	1400	58
/	18S rRNA	F: TATACGCTAGTGGAGCTGGAA<br>R: GGGGAGGTAGTGACGAAAAAT	136	55
ncbi_113815990	dihydropyrimidinase-like isoform X3	F: AGGGACAGGGCTAATGCTC<br>R: GGTTGACACCTCCACTTCTA	163	56
ncbi_113825784	prohibitin	F: TAAAGGCTGTTGTGGCTCG<br>R: CCGAAGGTCAAATGGGTAA	148	56
ncbi_113824882	iroquois-class homeodomain protein IRX-2-like	F: CTCCTCGTCCAGCACAGAC<br>R: GGGTAAGCATTGGGTAGCG	165	57
ncbi_113829145	diacylglycerol kinase 1	F: CGAGGAGGGATGACCACTA<br>R: ACGCAGGTTTGCTGAAGTG	107	56
ncbi_113813376	tubulin alpha-3 chain-like	F: CTACCGCCTTCTGTTCCACC<br>R: TCCACGATTTCCTTACCGATT	102	56
ncbi_113819252	actin	F: TGTGATGGTCGGTATGGGT<br>R: ATGAGTACGACGAGTCTGG	165	55
ncbi_113813611	troponin I	F: ACAATGAGCTCCGTGTTGC<br>R: TTCTATTACTACGTCTCGGATG	100	57
ncbi_113829244	MAM and LDL-receptor class A domain-containing protein 2-like	F: GGCTGGGAATGCGTGGTA<br>R: GCCCTGGATGATGGAAAT	133	55
ncbi_113820123	glyceraldehyde-3-phosphate-dehydrogenase	F: GACATCCGCTCCTCCATCT<br>R: CCCTCCCTTCCTCACATCC	185	56
ncbi_113807541	immune-associated nucleotide-binding protein 13-like	F: AGCTGGTAAGACTAGGCTGT<br>R: CTCATCTACCAGCAACCC	166	56
ncbi_113823028	alpha-(1,6)-fucosyltransferase-like	F: AATGGAGTTTAGCACGGGTAC<br>R: TTTGGGTTTGTCGGTGTCG	139	57
