# Supplementary material for: Two Independently Comparative Transcriptome Analyses of Hemocytes Provide New Insights into Understanding the Disease-Resistant Characteristics of Shrimp against Vibrio Infection
Source: Biology (Basel). 2023 Jul 10;12(7):977. doi: 10.3390/biology12070977 (PMC10376663; doi:10.3390/biology12070977)
Supplement: Supplementary file 1 [file biology-12-00977-s001.zip › Table S2 The identified DEGs from population 1.pdf]

Table S2. The identified DEGs from population 1

Gene ID	HcS1_ 1_fpkm	HcS1_ 2_fpkm	HcS1_ 3_fpkm	HcD1_ 1_fpkm	HcD1_ 2_fpkm	HcD1_ 3_fpkm	HcS1_mean	HcD1_mean	log2(fc)	PValue	FDR	Description
ncbi_113820920	0.713	0.17	0.302	0	0	0	0.395	0.001	-8.626	5.19E-05	3.76E-02	PREDICTED: structural maintenance of chromosomes protein 2-like [Crassostrea gigas]
MSTRG.35886	0.719	7.661	11.549	0.095	0	0.092	6.643	0.062	-6.736	3.30E-05	2.83E-02	hypothetical protein C7M84_023505 [Penaeus vannamei]
ncbi_113827946	2.778	7.205	8.993	0.229	0.339	1.002	6.325	0.523	-3.595	2.19E-07	4.18E-04	-
ncbi_113815624	0.5	0.785	0.841	0.085	0.094	0.064	0.709	0.081	-3.129	1.67E-05	1.54E-02	PREDICTED: vang-like protein 2 [Hyalella azteca]
ncbi_113827942	1.515	3.897	7.218	0.194	0.389	1.039	4.210	0.541	-2.961	3.24E-06	3.71E-03	-
MSTRG.1662	8.129	3.672	5.505	0.757	1.721	1.647	5.769	1.375	-2.069	1.09E-06	1.72E-03	APC membrane recruitment protein 1-like [Portunus trituberculatus]
MSTRG.226	12.58	16.344	13.035	6.309	6.224	7.619	13.986	6.717	-1.058	3.90E-06	4.19E-03	hypothetical protein EAI_04788, partial [Harpegnathos saltator]
ncbi_113812130	71.934	68.554	60.711	109.904	112.378	106.101	67.066	109.461	0.707	9.31E-05	4.99E-02	PREDICTED: 4-coumarate--CoA ligase 1 isoform X1 [Strongylocentrotus purpuratus]
ncbi_113824615	186.843	195.157	176.746	305.27	398.664	416.743	186.249	373.559	1.004	5.11E-08	1.25E-04	spermatogonial stem-cell renewal factor [Fenneropenaeus chinensis]
ncbi_113826008	10.15	13.107	10.29	24.94	29.114	17.654	11.182	23.903	1.096	5.50E-05	3.78E-02	-
MSTRG.4583	7.871	10.311	15.885	25.783	37.003	64.872	11.356	42.553	1.906	1.71E-05	1.54E-02	arasin-like protein [Penaeus vannamei]
ncbi_113807541	57.456	54.471	42.108	54.324	318.286	220.93	51.345	197.847	1.946	6.47E-05	3.97E-02	PREDICTED: immune-associated nucleotide-binding protein 13-like [Oreochromis niloticus]
ncbi_113806635	14.755	36.628	10.343	79.203	39.368	135.656	20.575	84.742	2.042	6.35E-05	3.97E-02	-
ncbi_113817613	3.657	9.375	3.13	12.939	32.139	30.986	5.387	25.355	2.235	2.05E-06	2.71E-03	PREDICTED: E3 ubiquitin-protein ligase TRIM32 [Python bivittatus]
ncbi_113824399	93.533	87.388	65.264	130.927	680.224	355.612	82.062	388.921	2.245	1.57E-06	2.25E-03	Septin-4-like protein [Daphnia pulex]
MSTRG.19559	4.607	4.705	8.075	6.926	45.03	32.355	5.796	28.104	2.278	4.78E-05	3.73E-02	hypothetical protein C7M84_014536 [Penaeus vannamei]

ncbi_113813284	84.025	124.063	84.648	272.1	809.682	415.108	97.579	498.963	2.354	4.74E-10	2.03E-06	-
ncbi_113825938	3.23	5.677	1.33	3.03	33.817	40.5	3.412	25.782	2.918	6.86E-05	4.06E-02	PREDICTED: uncharacterized protein LOC108677898 isoform X1 [Hyalella azteca]
ncbi_113800624	27.26	28.997	17.849	130.533	228.026	224.836	24.702	194.465	2.977	2.56E-23	4.39E-19	TUS1306 [Penaeus monodon]
ncbi_113813611	0.77	0.151	0.099	4.366	2.083	3.166	0.340	3.205	3.237	4.78E-05	3.73E-02	troponin I [Litopenaeus vannamei]
ncbi_113805465	0.966	0.529	0.488	12.268	3.291	3.255	0.661	6.271	3.246	1.10E-06	1.72E-03	myosin light chain 2 [Procambarus clarkii]
ncbi_113822686	1.284	0.165	0.49	11.51	4.681	6.559	0.646	7.583	3.552	2.58E-08	7.37E-05	myosin light chain [Marsupenaeus japonicus]
ncbi_113820123	3.804	6.28	5.72	32.214	55.636	118.83	5.268	68.893	3.709	4.11E-17	2.35E-13	glyceraldehyde-3-phosphate-dehydrogenase [Cherax cainii]
ncbi_113819252	0.308	0	0.056	5.688	0.95	1.58	0.121	2.739	4.497	1.65E-05	1.54E-02	actin 2 [Penaeus monodon]
ncbi_113816511	0.06	0.043	0.012	0.139	0.556	2.164	0.038	0.953	4.636	2.89E-06	3.54E-03	PREDICTED: myosin heavy chain, muscle-like isoform X4 [Hyalella azteca]
MSTRG.15220	0.068	0.262	0.215	10.783	0.38	10.883	0.182	7.349	5.338	1.10E-07	2.36E-04	Retrovirus-related Pol polyprotein from transposon 297,Retrovirus-related Pol polyprotein from transposon 17.6 [Mytilus coruscus]
ncbi_113823394	0	0	0.046	0.042	1.897	1.772	0.015	1.237	6.334	9.14E-05	4.99E-02	Protein CBG05275 [Caenorhabditis briggsae]
ncbi_113807016	0	0.015	0	0.046	0.229	1.029	0.005	0.435	6.442	7.18E-05	4.11E-02	PREDICTED: myosin heavy chain, muscle-like isoform X8 [Hyalella azteca]
ncbi_113818166	0	0	0	0.123	0.274	0.871	0.001	0.423	8.723	5.26E-05	3.76E-02	-
ncbi_113823028	0	0	0	1.46	5.039	2.399	0.001	2.966	11.534	3.25E-09	1.11E-05	PREDICTED: alpha-(1,6)-fucosyltransferase-like [Parasteatoda tepidariorum]
ncbi_113829244	0	0	0	9.114	7.289	14.557	0.001	10.320	13.333	2.70E-20	2.32E-16	PREDICTED: MAM and LDL-receptor class A domain-containing protein 2-like [Hyalella azteca]
