# Supplementary material for: Two Independently Comparative Transcriptome Analyses of Hemocytes Provide New Insights into Understanding the Disease-Resistant Characteristics of Shrimp against Vibrio Infection
Source: Biology (Basel). 2023 Jul 10;12(7):977. doi: 10.3390/biology12070977 (PMC10376663; doi:10.3390/biology12070977)
Supplement: Supplementary file 1 [file biology-12-00977-s001.zip › Table S3 The identified DEGs from population 2.pdf]

Table S3. The identified DEGs from population 2

Gene ID	HcS2-1_fpkm	HcS2-2_fpkm	HcS2-3_fpkm	HcS2-4_fpkm	HcD2-1_fpkm	HcD2-2_fpkm	HcD2-3_fpkm	HcD2-4_fpkm	HcS2_mean	HcD2_mean	log2(fc)	PValue	FDR	Description
ncbi_113815990	10.213	12.548	16.482	5.415	3.158	4.893	2.051	3.749	11.165	3.463	-1.689	3.10E-06	1.84E-02	PREDICTED: dihydropyrimidinase-like isoform X3 [Hyalella azteca]
ncbi_113825784	12.613	19.227	9.54	13.806	7.178	3.88	4.288	4.886	13.797	5.058	-1.448	1.37E-05	4.73E-02	prohibitin [Litopenaeus vannamei]
ncbi_113824882	4.686	5.927	5.405	4.87	2.134	1.596	2.681	1.827	5.222	2.060	-1.342	3.20E-06	1.84E-02	PREDICTED: iroquois-class homeodomain protein IRX-2-like [Limulus polyphemus]
ncbi_113829145	6.873	7.048	5.232	6.205	2.391	3.467	3.064	1.706	6.340	2.657	-1.255	9.34E-06	4.02E-02	PREDICTED: diacylglycerol kinase 1 [Ceratosolen solmsi marchali]
ncbi_113813376	1.746	1.442	1.167	1.348	2.718	6.186	12.265	6.076	1.426	6.811	2.256	8.27E-07	1.42E-02	PREDICTED: tubulin alpha-3 chain-like [Hyalella azteca]
